# Retraction: ‘Involvement of miR-4262 in paclitaxel resistance through the regulation of PTEN in non-small cell lung cancer’ (2019), by Sun *et al.*

**DOI:** 10.1098/rsob.230402

**Published:** 2023-11-03

**Authors:** Open Biology editorial team

**Keywords:** non-small cell lung cancer, paclitaxel, miRNA-4262, PTEN, PI3 K/Akt pathway

*Open Biol.* **9**, 180227 (Published online 24 July 2019). (https://dx.doi.org/10.1098/rsob.180227)

Following an investigation, we have found duplicates in the figures presented (notably fig. 2. (f) ‘A transwell migration assay was conducted to analyse the migratory capacity of A549 and H1299 cells transfected with the miR-4262 mimics or inhibitor at 18 h.’). This paper has been identified among of a set of papers that present significant text and data duplication and therefore the data reported in this article are unreliable. The authors have not been able to supply the raw data as requested, thus, the editors consider the conclusions of this article to be invalid. The authors have not responded to correspondence about this retraction.

The image integrity standards and policies for the journal can be found here: https://royalsocietypublishing.org/doi/10.1098/rsob.200165.

Jonathon Pines FRS

Editor-in-Chief, *Open Biology*.

